# HPV types in cervical cancer tissue in South Africa: A head-to-head comparison by mRNA and DNA tests

**DOI:** 10.1097/MD.0000000000008752

**Published:** 2017-11-27

**Authors:** Amir Rad, Sveinung Wergeland Sørbye, Greta Dreyer, Siri Hovland, Bente Marie Falang, Melanie Louw, Finn Egil Skjeldestad

**Affiliations:** a Department of Community Medicine, Faculty of Health Sciences, UiT The Arctic University of Norway; b Department of Clinical Pathology, University Hospital of North Norway, Tromsø, Norway; c Department of Obstetrics and Gynaecology, University of Pretoria, Pretoria, South Africa; d PreTect AS, Klokkarstua, Norway; e Department of Anatomical Pathology, University of Pretoria, Pretoria, South Africa.

**Keywords:** cervical cancer, DNA diagnostics, HPV-type distribution, mRNA diagnostics, prevalence

## Abstract

Accurate identification of human papillomavirus (HPV)-types in cervical cancer tissue may be important for tailoring tests for primary screening and types to be included in a vaccine. The aim of this study was to compare test-performance of a 45-type HPV deoxyribonucleic acid (DNA)-test with a 9-type HPV messenger ribonucleic acid (mRNA)-test in cervical cancer tissues.

In a case-series design 188 women with diagnosed cervical cancer during the period January 2008 to July 1, 2011 at the Gynaecological Oncology Unit, University of Pretoria, South Africa were recruited to the study. After cases with negative internal controls for DNA/mRNA detection (n = 18) and unconfirmed histology (n = 3) of cervical cancer were excluded, 167 women remained eligible for analysis. We compared 45 DNA-types detected through general primer (GP)5^+^/6^+^ polymerase chain reaction (PCR) and reverse line blot (RLB) genotyping with a modified version of the mRNA test PreTect HPV-Proofer detecting 9 genotypes (16, 18, 31, 33, 35, 45, 51, 52, 58).

Histological types were 92.2% squamous cell carcinoma, 4.8% adenocarcinoma, and 3.0% adenosquamous carcinoma. Overall, HPV was detected in 95.2% (159/167) of specimens. The DNA- and mRNA tests each rendered 153/167 (91.6%) HPV positive results. When restricting the analysis to the 9 high-risk HPV-types included in the mRNA test, 91.6% (153/167) and 88.0% (147/167) were positive by the mRNA- and DNA-tests (*P* = .28), respectively. After hierarchical categorization of 9 comparable types, we found concordance in 66 of 67 specimens for HPV16, 25 of 27 specimens for HPV18, 19 of 21 specimens for HPV45, and only in 33 of 45 for HPV31, 33, 35, 51, 52, 58. The positivity rate for the HPV types 16, 18, and 45 and the positivity rate for HPV 16, 18, 45, 33 and 35 by both tests was 66% to 68% and 80% to 83%, respectively.

Overall and when considering established high-risk types, the mRNA test has at least as high detection rate as the DNA test. The mRNA test can be an appropriate research tool to describe causative HPV-types in cervical cancer tissue for health care planning purposes.

## 1 Introduction

Cervical cancer ranks in women as the fourth most common cancer worldwide.^[1]^ In South Africa, cervical cancer is the most frequent cancer in women aged 15 to 44 years and the second among women of all ages.^[2]^

Human papillomavirus (HPV) infection is the necessary, but not sufficient, cause for cervical cancer.^[3–5]^ Persistent HPV infection is the most important risk factor for cervical cancer.^[6]^ HPV-targeted screening programs and HPV vaccination are implemented in many countries to reduce cervical cancer incidence, morbidity, and mortality.^[7]^ Knowledge of HPV type distribution in cervical precancerous and cancer histology is important to prioritize HPV types in future HPV prophylactic vaccines and HPV-based screening tests.^[8]^

Evaluation of the carcinogenic properties of HPV lacks longitudinal studies with cervical cancer as endpoint.^[9,10]^ Meta-analysis on prevalence of HPV types detected in cervical cancer reutilize the same studies,^[5,8,11,12]^ which lack consistency in HPV detection methods applied, number of HPV types targeted, and often validation of histological diagnoses are missing. In addition, most studies report all HPV types identified without a hierarchical approach to the types that are major drivers in the oncogenesis. This may overestimate the role of the low prevalent types, which often are appearing as coinfections in cervical cancer tissue. Knowledge on the biological mechanisms of HPV carcinogenicity is limited to basic research applied mostly to HPV16- and less to HPV18-infected cell lines,^[13–15]^ while evidence from basic research on the carcinogenic properties of other HPV types is lacking.

HPV tests differ in their clinical performance, sensitivity, and specificity.^[16–20]^ The characteristics of HPV tests are different in targeted nucleic acid (deoxyribonucleic acid [DNA] or ribonucleic acid [RNA]), targeted genes in HPV genome, and the ability of separate genotyping.^[21]^ The general primer (GP)5+/6+ polymerase chain reaction (PCR)-enzyme immunoassay (EIA) (polymerase chain reaction-*reverse* line blot) assay targets the L1 region of HPV DNA with no ability to report genotypes separately, while PreTect HPV-Proofer targets E6/E7 regions of HPV messenger ribonucleic acid (mRNA) and can detect types 16, 18, 31, 33, and 45, separately.^[21]^

DNA-based HPV tests detect the presence of HPV at DNA level and not necessarily the transcriptional and translational activity of the HPV DNA. The oncogenic activity of HPV type 16 is known to be through the expression of viral genes E6 and E7, following inactivation of cell tumor suppressor proteins p53 and protein retinoblastoma (pRB).^[22]^ The E6 and E7 gene expression from HPV types 16, 18, 31, 33, and 45 has been confirmed in the majority of cervical carcinomas.^[23]^ The mRNA-based HPV test detects the E6 and E7 oncogenic expression of HPV and it is based on the real-time multiplex nucleic acid sequence-based amplification (NASBA) assay called PreTect HPV-Proofer.^[24]^ On the other hand, there are several methods to test the presence of HPV DNA including PCR, reverse line blot (RLB) sequencing, in situ hybridization (ISH) and EIA, with PCR being the most commonly applied method for HPV DNA analysis. The PCR-based tests are using either consensus PCR primers that can cover a range of DNA types or type-specific PCR primers that work for specific genotypes. Depending on PCR primers, the size of the PCR-amplified fragment differs; for instance, the amplified fragment for MY09/11 is about 450 base pairs (bp) while the GP5/6 fragment size is approximately 140 bp.^[25]^ The short PCR fragment primers (SPF10), which were developed for universal detection of HPV, target only 65 bp of the L1 open reading frame (ORF) in at least 43 HPV genotypes.^[26,27]^ The SPF10 primers are more sensitive than other primers, especially when multiple HPV genotypes are present.^[27,28]^ It is noticeable that as the applied primers in HPV DNA test have fewer base pairs, the ability of the test to detect the presence of HPV DNA in tumor tissues and, consequently the test sensitivity, increases. Conversely, the specificity of these tests drops and it becomes less informative on the oncogenic properties. The DNA-based HPV tests detect the HPV viral DNA presence, which might be in transient phase and not active oncogenes while mRNA test positivity implies continuous expression of the viral E6 and E7 oncogenes.

The aim of this study was to compare the test-performance of a 45-type HPV DNA-test with a nine-type HPV mRNA-test in cervical cancer tissues.

## 2 Materials and methods

This study was performed in collaboration between the Institute of Community Medicine (ISM) and Department of Clinical Pathology, Faculty of Health Sciences, University of Tromso, Norway; PreTect AS, Klokkarstua, Norway; and the Gynecologic Oncology Unit, Departments of Obstetrics and Gynaecology and Anatomical Pathology, University of Pretoria, South Africa. The Research Ethics Committee of the Faculty of Health Sciences of the University of Pretoria reviewed and approved the study protocol (27/2008, 108/2008, 189/2012). All the participants gave written informed consent. At the time of presentation for the evaluation and staging of disease, tissue biopsies were taken for histological confirmation of the diagnosis of invasive epithelial cervical cancer and HPV analysis. Two adjacent punch biopsies were taken at the 3 o’clock and 9 o’clock positions. One biopsy from each position was preserved in formalin and sent to the Department of Anatomical Pathology at the University of Pretoria for histological diagnosis. Two pathologists reviewed all histological diagnosis until consensus was reached.

The second biopsy from each position was preserved in a standard commercially available methanol-buffer solution, PreTect TM (PreTect AS) and shipped to Norway for HPV DNA and mRNA analyses. These biopsies were cut in small pieces on a cold metal block using a scalpel and transferred to a microcentrifuge tube prior to addition of 1 mL lysis buffer (NucliSens, BioMerieux, France), followed by homogenization for 30 seconds using a pellet pestle and incubation at 37°C for 30 minutes. Total nucleic acids (DNA/RNA) were extracted using NucliSENS miniMAG (BioMerieux, 200297, Boxtel, The Netherlands) according to the manufacturer's instructions and kept at −70°C prior to DNA/mRNA testing performed on the same extracts. All laboratory testing was performed blindly.

Human papillomavirus DNA analysis, testing for 39 individual types (HPV 6, 11, 16,18, 26, 30, 31, 33, 34, 35, 39, 40, 42, 43, 44, 45, 51, 52, 53, 54,55, 56, 57, 58, 59, 61, 64, 66, 67, 68, 69, 70, 71, 72, 73, 81, 82/MM4, 82/IS39, and CP6108) and 6 rare HPV types (HPV32, 83, 84, 85, 86, and JC9710) as a pool, was performed on GP5+/6+ polymerase chain reaction products using RLB assay.^[29,30]^ Polymerase chain reaction toward the B-globin gene was included as DNA control for all HPV-negative samples.

Human papillomavirus mRNA E6/E7 analysis, testing for 9 individual HPV types (HPV 16, 18, 31, 33, 45, 35, 51, 52, 58) was performed using an extended version of PreTect HPV-Proofer, a diagnostic kit for the qualitative detection and direct typing of E6/E7 mRNA from 5 HPV types^[16,18,31–33]^ plus 4 additional HPV types (35,51,52,58). The kit is based on real-time NASBA technology combining nucleic acid amplification and simultaneous detection with specific Molecular Beacon probes. NASBA is an enzymatic 1-step amplification process that is able to specifically amplify RNA in a double-strand DNA (dsDNA) background under isothermal conditions (41°C). By using an RNA T7-polymerase promoter to generate multiple RNA products at 41°C, double-stranded DNA is not denatured and consequently not amplified, hence the presence of genomic dsDNA will not cause false positives.^[31]^

Intrinsic sample control (ISC) directed against mRNA from a human housekeeping gene is included in the kit to assess specimen quality and reveal possible factors that may inhibit the amplification, hereby monitoring the entire test process. Standardized artificial oligonucleotides corresponding to the respective viral sequences were used as positive controls for each of the HPV types and water as negative control. The PreTect Analysis Software (PAS, PreTect AS) performed all assay validation, where all controls and specimen ISC results have to be valid to report an HPV result.

A total of 188 patients with invasive cervical cancer referred to the gynaecologic oncology unit at the University of Pretoria during the period January 1, 2008 to July 31, 2011 were recruited to the study. We excluded women without validated histological diagnosis of cervical cancer (n = 3) and samples with low quality of genomic material by using ISC (n = 18). The final study population comprised 167 patients.

All data analyses were done in SPSS, version 24.0. We applied a 2-tailed 2-proportion Z-test to compare positivity rates between the tests with significance level *P* < .05.

We applied a hierarchical approach where one type is counted only once by decreasing prevalence order of HPV types in our own data.

## 3 Results

The majority of patients (77.2%) were older than 40 years (range 25–89 years), and 41.3% (69/167) were HIV positive. Histology results showed 92.2% (154/167) cases of squamous cell carcinoma (SCC), 4.8% adenocarcinoma, and 3.0% adenosquamous carcinoma. Among women less than 40 years, 81.6% (31/38) were HIV-positive. All women in this age group had SCC. Among women older than 40 years, 29.5% (38/129) were HIV-positive, and 90% (116/129) were diagnosed with SCC (Table 1).

**Table 1:**
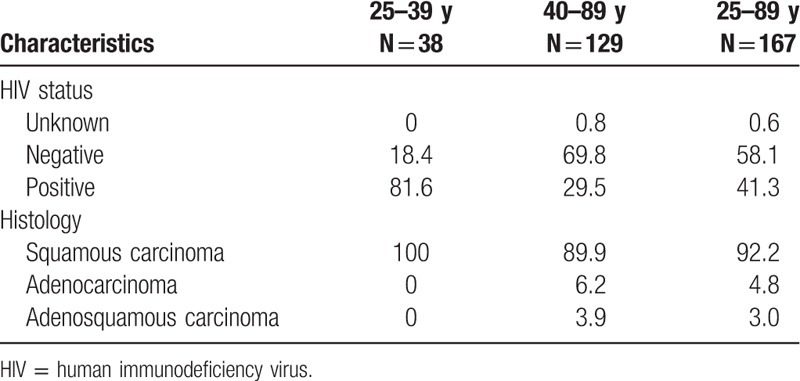
Study population characteristics, HIV status, and histology by age (%).

Considering both the DNA- and mRNA test results, 95.2% (159/167) of the specimens were HPV positive in at least 1 test, while the DNA- and the mRNA-tests each rendered 91.6% (153/167) HPV positive results. In 7.8% (13/167) specimens HPV were detected only by the DNA test and similarly in 7.8% (13/167) specimens, HPV were detected only by mRNA test. There were 11 double infections detected by mRNA analysis and 4 double infections detected by DNA analysis.

When analyzing the 9 most prevalent HPV types hierarchically (16>18>45>35>33>52>31>58>51), 91.6% (153/167) were positive by the mRNA test and 88.0% (147/167) by the DNA-test (*P* = .28). In total, 83.8% (140/167) were positive for the same HPV type by both tests, while there were 26 discordant results. We found concordance in 66 of 67 cases of HPV16, 25 of 27 cases of HPV18, 19 of 21 cases of HPV45, 15 of 18 cases of HPV35, and 18 of 27 cases of HPV types 33, 52, 31, 58, and 51 collectively. HPV types 30, 56, 69, 73, 82 were not included in the mRNA test, which added 6 more positive cases by the DNA test (Table 2, lower panel).

**Table 2:**
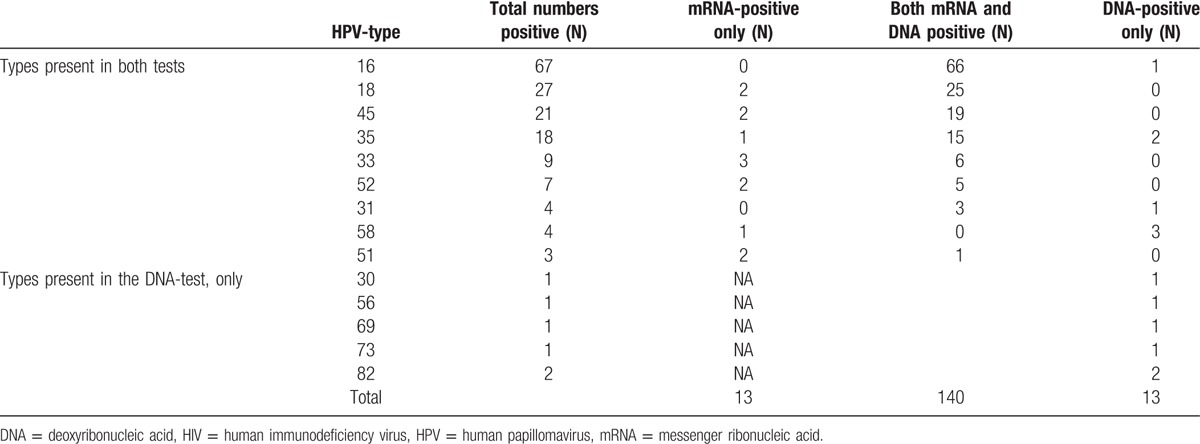
Concordant and discordant pairs in DNA/mRNA analysis of type-specific HPV detection.

Among the HPV-negative cases, 8 biopsies were negative in both the tests. In addition, 6 biopsies were negative only by the DNA test and 6 biopsies were negative only by the mRNA test.

The positivity rate for the HPV types 16, 18, and 45 and the positivity rate for HPV 16, 18, 45, 33, and 35 by both tests summarized to 66% to 68% and 80% to 83%, respectively. Thirty-eight women were less than 40 years of age, among whom 31 women were HIV-positive. Twenty-nine of these 31 HIV infected women tested positive for HPV by both tests; 29 and 26 out of 31 HIV positive women were positive for the 9 high risk types by the mRNA- and the DNA-test, respectively. Table 3 displays a more complete comparison of the concordance and discordance in DNA/mRNA analyses among HIV negative and positive women. Positivity rates of type-specific HPV by DNA- and mRNA-tests did not differ in any of the comparisons (data not shown).

**Table 3:**
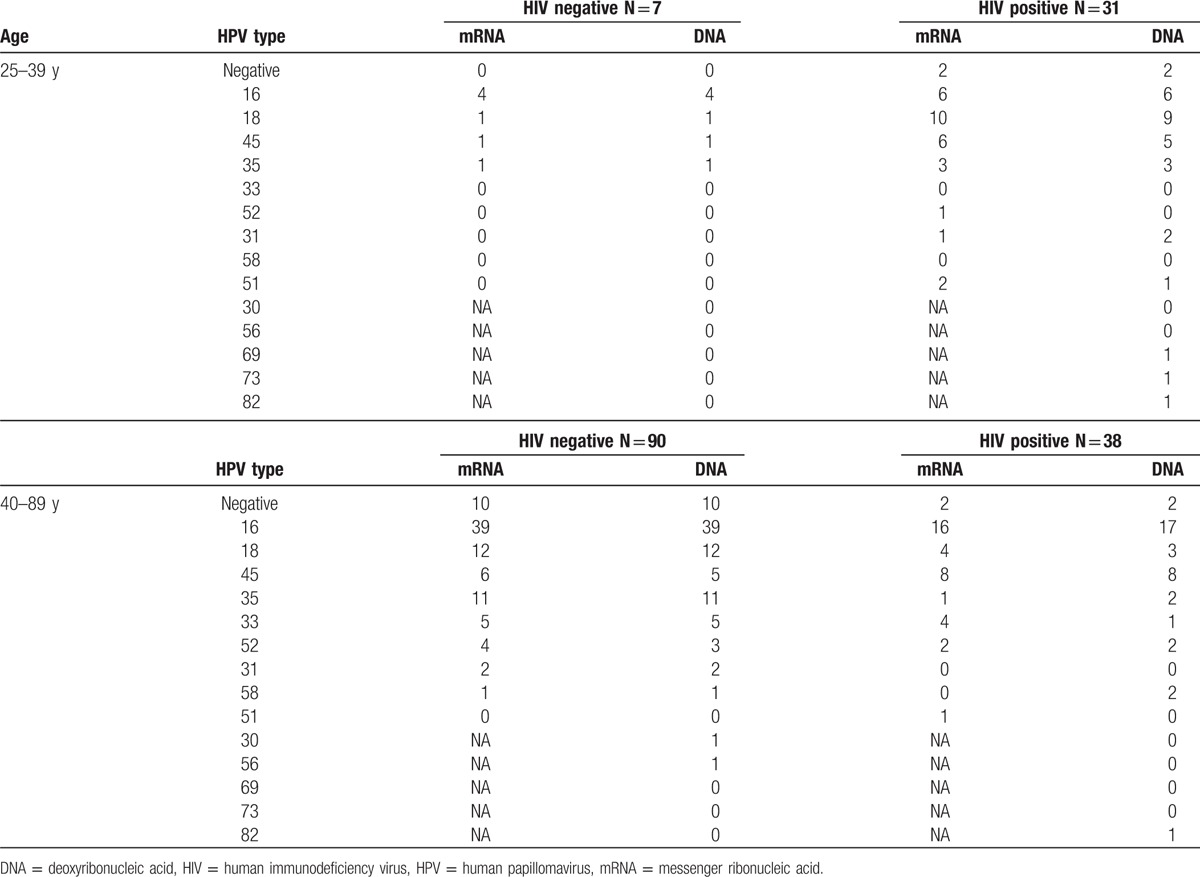
Comparison in DNA/mRNA analysis of type-specific HPV detection in HIV negative/positive women stratified by age.

## 4 Discussion

In this case-series of 167 women diagnosed with cervical cancer, 95.2% (159/167) were HPV positive in at least 1 test. There were no differences in overall comparison with types detected by the 45 types DNA test (91.6%) and the 9 types mRNA test (91.6%).

### 4.1 Overall positivity rate

In most prevalence studies of HPV detection in cervical cancer tissue, 2 to 5 different methods are used to diagnose the virus. A valid positive test result is based on at least 1 test being positive. Our overall 95.2% HPV positivity rate was higher than in another African study (Ghana, Nigeria, and South Africa (90.4%))^[34]^ and similar to what has been reported from a European study that tested for HPV DNA in cervical cancer tissue.^[32]^ Another study from Norway found an overall 97% HPV positivity rate in tissue from squamous cell carcinoma,^[23]^ while the type-specific PCR primers, consensus Gp5+/6+ PCR primers for HPV DNA, and 8-types E6/E7 mRNA test had equal 92% positivity rates.^[23]^ Similarly, a study from India reported an overall 91.7% positivity rate of HPV in cervical cancer specimens with no difference in positivity rate between MY09/11 L1 consensus PCR applied HPV DNA test and the PreTect HPV-Proofer (5 types).^[35]^ A meta-analysis summarizing results from case-series of HPV prevalence in cervical cancer tissue, regardless of method and number of methods used, demonstrated overall 87% HPV DNA positivity, reaching 94% in cervical cancer specimens from Africa.^[8]^

### 4.2 HPV type distribution

Our analyses are based on a hierarchical approach where each type is counted only once by order of decreasing prevalence. We found the same order of prevalence as summarized by Smith et al ^[8]^ in 5 studies from Africa, except for HPV 33 and HPV 52. Smith et al^[8]^ counted each type more than once if they occurred as double/tripled infections. However, we found a lower prevalence of HPV 16, similar prevalence of HPV 18, and higher prevalence for HPV 45, 33, and 52 than displayed by Smith et al^[8]^ and in another study from Ghana, Nigeria, and South Africa.^[34]^ Compared with a European study on prevalence of HPV types in cervical cancer tissue, we again found a lower prevalence of HPV16, and relatively higher prevalence of HPV 45, 35, and 52.^[32]^

### 4.3 Prevalence, persistency, and progressive ability

Evaluation of the carcinogenic properties of HPV suffers from the lack of long-term prospective studies with cervical cancer as endpoint.^[9,10]^ Most of the reviews and meta-analysis considered prevalence from case-control or case-series studies.^[5,8,11,12]^ Although HPV types may differ by order of magnitude in risk for cervical cancer,^[36]^ the International Agency for Research on Cancer (IARC) did not rank the HPV types according to this risk, except for types 16 and 18. They simply concluded that HPV types 16, 18, 31, 33, 35, 39, 45, 51, 52, 56, 58, and 59 are carcinogenic in the human cervix.^[36,37]^

In cervical carcinogenesis, genotype-specific HPV persistence is associated with higher risk of cervical cancer than transient HPV infection.^[10,38–40]^ Longitudinal prospective studies showed that some of the HPV types that are classified by the IARC and other above-mentioned studies as high risk or carcinogenic, had no or little potentiality for progression to high-grade cervical lesions and cancer.^[10,41–45]^

HPV types 16, 18, and 45 have been detected more frequently in invasive cervical cancer (ICC) than cervical intraepithelial neoplasia grade 3 (CIN3) cases, suggesting differences in type-specific risks for progression and the necessity of treatment for cervical lesions related to these types.^[8,32,33]^ This, together with the narrow median age differences between CIN3 and SCC in women diagnosed with HPV types 16, 18, and 45, indicates the progressive nature of HPV types 16, 18, and 45.^[32]^

### 4.4 HPV types distribution among HIV positive/negative women

In this study, similar to other studies from Mozambique,^[46]^ Kenya,^[47]^ and South Africa,^[47]^ we did not detect significant differences in positivity rates of HPV types by HIV status. Since cell-mediated immunity is crucial in clearing HPV infection and for regression of cervical lesions,^[48]^ we expected a different HPV-type distribution. Among immunocompromised women “low risk types” could become more “oncogenic,” but our results did not confirm such a theory. Lack of knowledge on the time of HIV acquisition is another difficulty in determining the oncogenic potential of HPV types by HIV status.^[47]^ In case the HIV infection took place after HPV infection and, especially, in the last years before cervical cancer development, the HIV-associated immune impairments would not affect the responsible HPV type.^[47]^ Moreover, it is supposed that micronutrient deficiency and chronic infections in African countries may also suppress the immune system and, consequently, fade the association between type-specific HPV infection and HIV status.^[49]^

### 4.5 Strengths

We consider the application of NASBA technology as a strength in mRNA detection method. This technology amplifies RNA under isothermal conditions, which avoids denaturing and, in turn, amplification of double stranded DNA. Therefore, the false positives from the presence of genomic dsDNA in the background of mRNA may be prevented.^[31]^ The usage of 2 different methods for HPV detection, together with the high concordance (84%) in type detection between methods, is considered another strength.

In addition, we consider the hierarchical approach in performing analyses for multiple infection cases as a strength. The hierarchical analysis of single infections avoids overestimation of the less prevalent types in cervical cancer specimen. The results from hierarchical studies provided more accurate information on the role of HPV16/18 compared with other oncogenic HPV types for the risk of CIN3 and cancer.^[43,45]^

### 4.6 Limitations

In a global perspective, our sample size may be considered a limitation,^[8,32]^ however, from a regional or national perspective, our sample size is above average of published studies. Some HPV types that tested positive using the DNA test were not covered by the mRNA test and thus could not be confirmed as carcinogenic. This could be a limitation for the mRNA detection and also type specific comparisons. However, these HPV-types are considered to have low oncogenic properties.

## 5 Conclusion

Overall and when considering established high-risk types, the mRNA test has at least as high a detection rate as the DNA test. The mRNA test can be an appropriate research tool to describe causative HPV-types in cervical cancer tissue for health care planning purposes.

## Acknowledgments

The authors thank Runi Rogers, Frank Karlsen, and Hanne Skomedal who initiated the study with the South-African study team.
